# Comparison of Clinical Safety and Outcomes of Early versus Delayed Laparoscopic Cholecystectomy for Acute Cholecystitis: A Meta-Analysis

**DOI:** 10.1155/2014/274516

**Published:** 2014-07-14

**Authors:** Min-Wei Zhou, Xiao-Dong Gu, Jian-Bin Xiang, Zong-You Chen

**Affiliations:** Department of General Surgery, Huashan Hospital, Fudan University, Shanghai 200040, China

## Abstract

*Objective.* To compare the clinical safety and outcomes of early laparoscopic cholecystectomy versus delayed laparoscopic cholecystectomy for acute cholecystitis. *Methods.* Pertinent studies were selected from the Medline, EMBASE, and Cochrane library databases, references from published articles, and reviews. Seven randomized controlled trials (early laparoscopic cholecystectomy versus delayed laparoscopic cholecystectomy) were selected. Conventional meta-analysis according to Cochrane Collaboration was used for the pooling of the results. *Results.* Seven trials with 1106 patients were included. There was no significant difference between the two groups in terms of bile duct injury (Peto odds ratio 0.49 (95% confidence interval 0.05 to 4.72); *P* = 0.54) or conversion to open cholecystectomy (risk ratio 0.91 (95% confidence interval 0.69 to 1.20); *P* = 0.50). The total hospital stay was shorter by 4 days for early laparoscopic cholecystectomy (mean difference −4.12 (95% confidence interval −5.22 to −3.03) days; *P* < 0.00001). *Conclusion.* Early laparoscopic cholecystectomy during acute cholecystitis is safe and shortens the total hospital stay.

## 1. Introduction

Acute cholecystitis occurs most commonly due to obstruction of the cystic duct with gallstones (cholelithiasis) and is among the most common acute abdomen diseases in emergency room. About 5–25% of the adult Western population have gallstones [1], and some people may become symptomatic every year.

First performed in 1985 by Dr Erich Mühe [2], laparoscopic cholecystectomy (LC) has now replaced open cholecystectomy (OC) as the first choice of treatment for gallstones and inflammation of the gallbladder unless contraindications are found with the laparoscopic approach [3–5]. With the development in laparoscopic skill and equipment, early LC has been reported as having significantly lower complication rates than early OC [6]. However, the timing of LC still remains controversial regarding the inflammation, edema, and adhesions of the acute course of disease. Nowadays, LCs for acute cholecystitis are now mainly performed after the acute episode occurrs, while conservative therapies, usually antibiotics, and delayed LCs are still common in many cases.

Several randomized clinical trials of comparisons of early laparoscopic cholecystectomy (ELC, performed within 7 days of onset of symptoms) with delayed laparoscopic cholecystectomy (DLC, performed at least 6 weeks after symptoms occurred) show that ELC could get more benefits in hospital stay and equally the same level of clinical safety, comparing with DLC [9, 10, 12–15]. However, the sample size was not big in previous clinical trials; recently Gutt et al. [7] performed a multicenter randomized controlled trial with a total of 618 patients (304 ELC, 314 DLC). Larger sample sizes are needed to confirm the accuracy of analysis results. We want to update systematic reviews. The aim of this study is to further assess the safety and outcomes of ELC versus DLC in people with acute cholecystitis and which kind of surgical procedure shows more benefits.

## 2. Methods

### 2.1. Literature Search

Search works were based on the databases of The Cochrane Library trials register, MEDLINE, and EMBASE. The search strategies involve articles comparing the outcomes of ELC with those of DLC between January 1988 and December 2013.

### 2.2. Inclusion Criteria

Randomized controlled trials comparing ELC with DLC of acute cholecystitis in adult patients were included despite of different status of language, blinding, and sample size. Quasirandomized controlled trials, nonrandomized studies, and retrospective studies were excluded. ELC must be performed within 7 days of onset of symptoms, while DLC were defined as initial conservative treatment followed by LC at least 1 week later.

All the included trials must report at least one of the primary outcomes (postoperative mortality; surgery-related morbidity such as bile duct injury, bile leak, reoperation rate, infection, and bleeding; complications during waiting time such as pancreatitis and recurrence of cholecystitis; conversion to OC) or secondary outcomes (operating time, hospital stay, and quality of life). The references of the included trials were further searched and identified.

### 2.3. Data Extraction and Statistical Analysis

Two authors (Min-Wei Zhou and Xiao-Dong Gu) independently identified all the data from the included studies. We used RevMan software (Version 5.2) for the meta-analysis in accordance with the recommendations of the Cochrane Collaboration.

The statistic of dichotomous outcomes was summarized by the risk ratio (RR) with 95% confidence interval (CI). Among these stats, Peto odds ratios (OR) were used when the proportion of people who developed the outcome was less than 1% (bile duct injury) [14]. For continuous variables, we calculated the mean difference (MD) with 95% CI. We considered the results to be statistically significant at the *P* < 0.05 level if the 95% confidence interval did not include the value 1.

We examined the forest plot to visually assess heterogeneity. According to Higgins's theory, heterogeneity was explored by the *χ* ^2^ test. The quantity of heterogeneity was measured by the *I* ^2^ statistic [16]. Intention to treat analysis was used in all analyses [17]. We followed the guidance of Gurusamy's study and used, namely, best-best analysis, worst-worst analysis, best- worst analysis, and worst-best analysis [14].

### 2.4. Assessment of Risk of Bias

In the design and process of randomized controlled trials, there might be some bias, which overestimates benefits of treatment [18–20]. We assessed the risk of bias by randomized sequence generation; allocation concealment; blinding of participants, personnel, and assessors; incomplete outcome data; selective outcome data according to the guidelines of The Cochrane Collaboration and the Cochrane Hepato-Biliary Group Module.

## 3. Results

### 3.1. Results of the Search

As shown in Figure 6, we identified a total of 833 references through the electronic searches of the Cochrane Central Register of Controlled Trials (CENTRAL) in The Cochrane Library (*n* = 192), MEDLINE (*n* = 416), and EMBASE (*n* = 225). We excluded 201 duplicates and 618 clearly irrelevant references through reading titles and abstracts. Fourteen references were retrieved for further assessment. Of the 14 references, 7 researches were excluded for the reason of unavailable full-text or mismatching inclusion criteria.

### 3.2. Quality of Assessment and Study Design

We included seven trials. Details of the included trials are shown in Table 1 and the Appendix The table summarizes the characteristics of all the included studies. A total of 1106 patients with acute cholecystitis were randomized to either ELC (*n* = 548) or DLC (*n* = 558). Gutt et al.'s study included patients treated in multiple centers, while others were single center studies.

We summarized the risk of bias in Table 2. Randomization methods were reported as computer generated [7, 9, 10, 12, 13], while Yadav et al.'s and Davila et al.'s studies did not describe the method of randomization sequence generation [8, 11]. Concealment of allocation was documented in two studies as use of sealed envelopes [9, 10, 12, 13]; it was not reported in the other two studies [8, 11]. Gutt et al. described their study as an open label trial. Owing to the nature of interventions used, none of the studies was blinded. All studies included were considered to be at high risk. Lo's study described dropouts and others did not.

### 3.3. Outcome Measures

#### 3.3.1. Primary Outcomes

*Mortality.* Gutt et al. reported the mortality was 0.3% in both of the groups (ELC 1/304; DLC 1/314) [7]. No participants in any of the other trials died.

**Figure 1:**
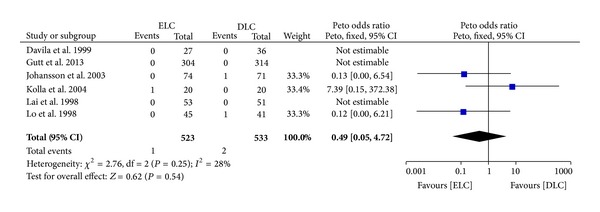
Meta-analysis of bile duct injury in ELC versus DLC. Peto odds ratio shown with 95% confidence intervals.

*Bile Duct Injury.* Five of the included studies reported bile duct injury, which was considered as one of the serious complications after LC. Stats showed there was no significant difference between the two groups (Peto OR 0.49 (95% CI 0.05 to 4.72); *P* = 0.54) (Figure 1). The rate of bile duct injury was 0.2% (1/523) in ELC group and 0.4% (2/533) in DLC group. There was no significant heterogeneity (*I* ^2^ = 28%; *P* = 0.25). 

**Figure 2:**
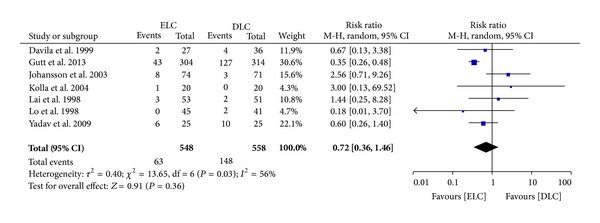
Meta-analysis of other complications in ELC versus DLC. Risk ratio shown with 95% confidence intervals.

*Other Complications.* Gutt et al.'s study presented that morbidity in ELC group was significantly lower than in DLC group (11.8% versus 34.4%). However, there was no significant difference between the two groups in general (RR 0.72 (95% CI 0.36 to 1.46); *P* = 0.36). There was heterogeneity among this trial with others (*I* ^2^ = 56%; *P* = 0.03) (Figure 2). Thus, we summarized the statistics by random-effects model. 

*Gallstone-Related Morbidity during Waiting Time.* During waiting periods of DLC group, 33 patients developed cholangitis and 3 got pancreatitis (cholangitis: 31/314; pancreatitis: 3/314, in Gutt et al.'s study). In five of the included studies, 17.5% (40/228) of patients in DLC group had recurred symptoms or were not relieved during the waiting period [9–13].

**Figure 3:**
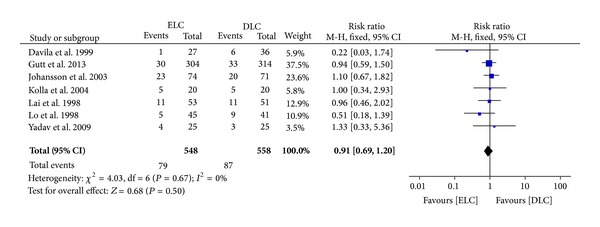
Meta-analysis of conversion to open cholecystectomy in ELC versus DLC. Risk ratio shown with 95% confidence intervals.

*Conversion to OC.* All the studies included conversion to OC. There was no significant difference in conversion to OC between the two groups (RR 0.91 (95% CI 0.69 to 1.20); *P* = 0.50) (Figure 3). The conversion rate was 14.4% (79/548) in ELC and 15.6% (87/558) in DLC group. There was no significant heterogeneity (*I* ^2^ = 0.00%; *P* = 0.67). We applied intention-to-treat analysis on conversion to OC. And no significant difference was shown.

#### 3.3.2. Second Outcomes

**Figure 4:**
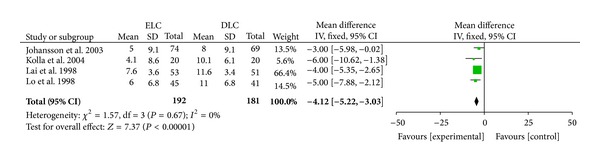
Meta-analysis of total hospital stay in ELC versus DLC. Risk ratio shown with 95% confidence intervals.

*Total Hospital Stay.* Five studies reported this outcome [7, 9, 10, 12, 13]. The mean and median were used in different studies. The median was used in the meta-analysis after imputing the standard deviation from the *P* value [10, 13]. Total hospital stay was significantly reduced in ELC group versus DLC group (MD −4.12 days (95% CI, −5.22 to −3.03); *P* < 0.00001) (Figure 4). There was no heterogeneity (*I* ^2^ = 0.00%; *P* = 0.67). The result remained if we excluded two median-using trials. In Gutt et al.'s trial, mean with interquartile was used and could not be applied to meta-analysis. Mean total length of hospital stay for immediate LC group was 5.4 days versus 10.0 days for DLC group. Length of hospital stay after cholecystectomy was about the same in both groups. Thus, there might be no major difference in surgical complications for different timing. 

**Figure 5:**
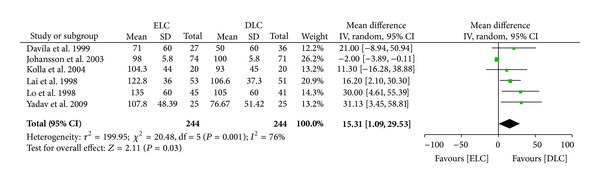
Meta-analysis of operation time in ELC versus DLC. Risk ratio shown with 95% confidence intervals.

**Figure 6:**
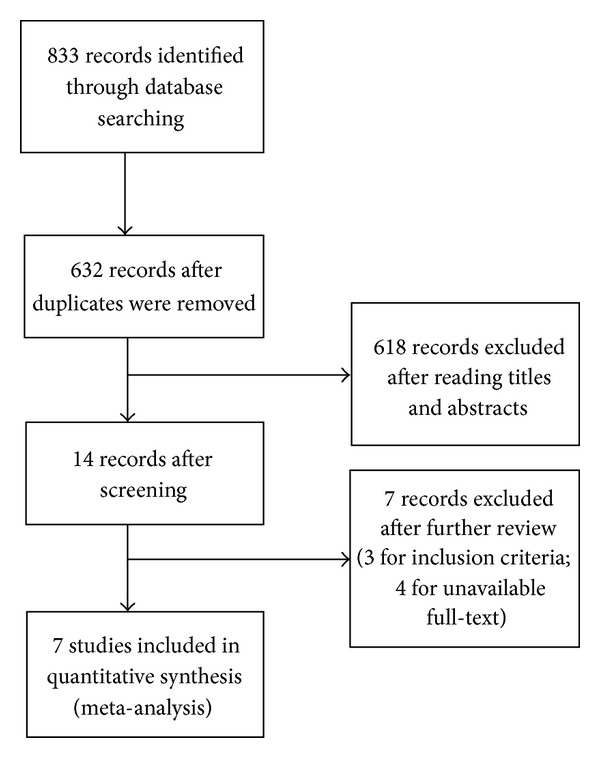
Study flow diagram.

*Operation Time.* There was significant heterogeneity among these trials with others (*I* ^2^ = 76%; *P* = 0.001). Thus, we summarized the statistics by random-effects model. There was a significant difference in operation time between the two groups (MD 15.31 (95% CI 1.09 to 29.53); *P* = 0.03) (Figure 5). Two trials reported the mean [9, 13] and three trials reported the median operating time [10–12]. The median was used in the meta-analysis. 

*Quality of Life.* None of the included trials reported quality of life.

## 4. Discussion

With the big sample of multicenter randomized trials [7], this meta-analysis of randomized clinical trials might become more convincing. No significant difference was found in the proportion of patients with complications (especially bile duct injury) or conversion to OC whether LC is performed at presentation with acute cholecystitis or it is performed more than 1 week after the symptoms settle. Early surgery was proved to have benefits of less hospital stay and lower risk of emergency surgery for nonresolved or recurrent symptoms which lead to a high rate of conversion to OC.

Mortality rate seems to keep an equal level of both early and late laparoscopic groups. This indicates the maturity of the techniques of LC and other conservative therapies. In some fatal cases, patients with severe acute cholecystitis exhibited organ dysfunction, which may lead to death.

The most common serious complications of LC are bile duct injury, which is fatal and necessary for reoperation [21]. Misidentification of the common bile duct as the cystic duct is the most common cause of bile duct injury [6]. Bile leakage is also a common postoperative complication. Five of the included articles reported bile leakage separately [7, 9, 10, 12, 13]. No significant difference was shown in both bile duct injury and bile leakage between two groups (bile duct injury: Peto OR 0.49 (95% CI 0.05 to 4.72, *P* = 0.54); bile leakage: OR 2.55 (95% CI 0.91 to 7.19, *P* = 0.08)). Of all the included studies, only that of Johansson et al. [10] involved routine performance of operative cholangiography. Routine operative cholangiography has been advocated to reduce the incidence of bile duct injury and major bile leakage. Although cholangiography performed through a divided but misidentified cystic duct definitely is too late to prevent the mishap from occurring [22], operative cholangiography seems to be necessary when common duct stones or bile duct injuries are suspected [23]. However, many surgeons did not conduct it for many kinds of reasons. It still needs further investigation.

It seems there was no significant difference in the other complications related to surgery between the two groups. And many other studies have the same result. Gutt et al. might be responsible for the heterogeneity. During the observation of postoperation periods, they took down all the complications and each patient could develop more than one, such as wound infection and fever and. When we excluded their trials, we could find no significant difference among the rest of the included studies and no heterogeneity (*I* ^2^ = 0.0%) as well. The rate of conversion to open surgery between the two groups was also at the same level. It is quite interesting that some included studies took the failure rate of conservative therapy in DLC group into consideration. Early studies indicated a high failure rate of conservative therapy when patients were waiting for delayed surgery [24, 25]. The failure mainly resulted from recurrent symptoms or progressive disease. 26% of DLC patients in Johansson et al.'s study [10], 16% in Lai et al.'s study [12], 20% in Lo et al.'s study [13], and 9% in Serralta et al.'s study [26] received emergency surgery instead of plan. This high rate of failure among patients with acute cholecystitis managed conservatively presents a strong and controversial issue if an aggressive immediate surgery was needed. Further valid and large-sample data are needed.

The optimal timing of surgery was still controversial. The analyzed trials lack a homogeneous definition of early surgical treatment. “Early” has been variably defined as anywhere from 24 h to 7 days after either the onset of symptoms or the time of diagnosis at hospital admission according to the included trials. Lee et al. [27] suggested the ideal threshold period should be within 72 h of symptom onset. In our review, a subgroup analysis showed no significant difference in the proportion of people with conversion to OC or complications between people operated on within four days and within seven days after the onset of symptoms. More data regarding the optimal timing is needed for further analysis.

The total hospital stay of the participants was shorter by four days with ELC than with delayed surgery. This may result from the more treatments and therapies. Surgery can clear the inflammation tissue and abscess much quicker than conservative therapy. Another reason was that patients in delayed group might receive extra treatment when the syndromes were not relieved or recurred. Gurusamy et al. [15] also mentioned that lack of blinding could be an important source of bias in the length of hospital stay. The total cost of early surgery seemed significantly lower than delayed group (€2919 versus €4262; *P* < 0.001) [7].

Within this meta-analysis, intention-to-treat analysis is really important. Proper intention-to-treat analysis could give a proper explanation to the statistics and different results.

## 5. Limitations of the Study

The overall quality of evidence was not very good, especially blinding. Studies that contributed to the meta-analyses in this review were at high risk of bias. Blinding is impossible to achieve for participants and care providers in this kind of studies. We should make some changes and pay more attention to blinding of assessors.

## 6. Conclusion

There was no significant difference between ELC and DLC in our primary outcomes. However, ELC during acute cholecystitis may reduce complications and shorten the total hospital stay. Despite this, high risk of bias may still influence these outcomes.

## Figures and Tables

**Table 1:** Baselines of included studies.

Studies	Year	Countries	Number of patients (ELC : DLC)	Average age	Females
Gutt et al. [7]	2013	Germany	618 (304 : 314)	56 years old	363 (58.7%)
Yadav et al. [8]	2009	Nepal	50 (25 : 25)	41 years old	38 (76%)
Kolla et al. [9]	2004	India	40 (20 : 20)	40 years old	32 (80%)
Johansson et al. [10]	2003	Sweden	145 (74 : 71)	57 years old	87 (60%)
Davila et al. [11]	1999	Spain	63 (27 : 36)	56 years old	45 (71.4%)
Lai et al. [12]	1998	Hong Kong, China	104 (53 : 51)	56 years old	66 (63.5%)
Lo et al. [13]	1998	Hong Kong, China	86 (45 : 41)	60 years old	39 (43.3%)

**Table 2:** Risks of bias in included studies.

Studies	Randomization sequence generation	Allocation concealment	Blinding	Incomplete outcome data	Selective reporting	Free from baseline imbalance
Gutt et al. [7]	+	+	−	+	+	+
Yadav et al. [8]	?	?	−	?	−	+
Kolla et al. [9]	+	+	−	+	+	+
Johansson et al. [10]	+	+	−	+	+	+
Davila et al. [11]	?	?	−	?	+	?
Lai et al. [12]	+	+	−	+	+	+
Lo et al. [13]	+	+	−	+	+	+

+: low risk of bias; −: high risk of bias; ?: unclear.

## Appendix

### Characteristics of the Included Studies

*See [7] (Gutt et al. 2013)*

*Methods.* They were multicenter randomized controlled trials.

*Participants.* All were adults with acute cholecystitis.

*Interventions.* Participants were randomly assigned to two groups:

- ILC (immediate laparoscopic cholecystectomy) group: < 24 hours of admission (*n* = 304);
- DLC group: surgery delayed by 7–45 days (*n* = 314).

*Outcomes.* The outcomes reported were mortality, morbidity (with scoring system), change of antibiotic therapy, conversion to open cholecystectomy, hospital costs, and total hospital stay.

*See [8] (Yadav et al. 2009)*

*Methods.* They were randomized controlled trials.

*Participants.* Adults were with acute cholecystitis. Exclusion criteria were as follows: (1) symptoms more than one week; (2) common bile duct stones or ductal dilatation; (3) contraindication for laparoscopic surgery; (4) people who refused to undergo laparoscopic surgery.

*Interventions.* Participants were randomly assigned to two groups:

- ELC group: as soon as possible (*n* = 25);
- DLC group: surgery delayed by 6–8 weeks (*n* = 25).

*Outcomes.* The outcomes reported were mortality, morbidity, conversion to open cholecystectomy, operation time, and total hospital stay.

*See [9] (Kolla et al. 2004)*

*Methods.* They were randomized controlled trials.

*Participants.* Adults were with acute cholecystitis. Exclusion criteria were as follows: (1) symptoms more than four days; (2) previous history of upper abdominal surgery; (3) contraindication for laparoscopic surgery; (4) common bile duct stones.

*Interventions.* Participants were randomly assigned to two groups:

- ELC group: < 24 hours of randomization (*n* = 27);
- DLC group: surgery delayed by 6–12 weeks (*n* = 36).

*Outcomes.* The outcomes reported were mortality, morbidity, conversion to open cholecystectomy, operation time, and total hospital stay.

*See [10] (Johansson et al. 2003)*

*Methods.* They were randomized controlled trials.

*Participants.* Adults were with acute cholecystitis. Exclusion criteria were as follows: (1) symptoms more than one week; (2) older than 90 years old; (3) bilirubin more than 3.5 mg/dL.

*Interventions.* Participants were randomly assigned to two groups:

- ELC group: < 7 days of diagnosis (*n* = 74);
- DLC group: surgery delayed by 6–8 weeks (*n* = 71).

*Outcomes.* The outcomes reported were mortality, morbidity, conversion to open cholecystectomy, operation time, and total hospital stay.

*See [11] (Davila et al. 1999)*

*Methods.* They were randomized controlled trials.

*Participants.* All were adults with acute cholecystitis.

*Interventions.* Participants were randomly assigned to two groups:

- ELC group: < 7 days of diagnosis (*n* = 27);
- DLC group: surgery delayed by 2 months (*n* = 36).

*Outcomes.* The outcomes reported were mortality, morbidity, conversion to open cholecystectomy, and total hospital stay.

*See [12] (Lai et al. 1998)*

*Methods*. They were randomized controlled trials.

*Participants.* Adults were with acute cholecystitis. Exclusion criteria were as follows: (1) symptoms more than one week; (2) previous history of upper abdominal surgery; (3) contraindication for laparoscopic surgery; (4) common bile duct stones; (5) acute pancreatitis or acute cholangitis.

*Interventions.* Participants were randomly assigned to two groups:

- ELC group: < 24 hours of randomization (*n* = 53);
- DLC group: surgery delayed by 6–8 weeks (*n* = 36).

*Outcomes.* The outcomes reported were mortality, morbidity, conversion to open cholecystectomy, operation time, and total hospital stay.

*See [13] (Lo et al. 1998)*

*Methods.* They were randomized controlled trials.

*Participants.* Adults were with acute cholecystitis. Exclusion criteria were as follows: (1) symptoms more than one week; (2) previous history of upper abdominal surgery; (3) contraindication for surgery; (4) more than three days of admission; (5) uncertainty about diagnosis; (6) peritonitis; (7) pregnancy.

*Interventions.* Participants were randomly assigned to two groups:

- ELC group: < 72 hours of admission (*n* = 45);
- DLC group: surgery delayed by 8–12 weeks (*n* = 36).

*Outcomes.* The outcomes reported were mortality, morbidity, conversion to open cholecystectomy, operation time, total hospital stay, return to normal activities, and return to work.
